# Draft Genome Sequence of the Marine *Flavobacteriaceae* sp. Strain LSUCC0859

**DOI:** 10.1128/mra.00186-22

**Published:** 2022-05-02

**Authors:** Holly R. D. Stapelfeldt, Shelby J. Barnes, Michael W. Henson, J. Cameron Thrash

**Affiliations:** a Department of Biological Sciences, University of Southern California, Los Angeles, California, USA; b Department of Geophysical Sciences, University of Chicago, Chicago, Illinois, USA; Montana State University

## Abstract

A new marine *Flavobacteriaceae* sp. strain, LSUCC0859, was isolated off the coast of Louisiana with artificial seawater via high-throughput dilution-to-extinction (DTE) cultivation. The 2,168,862-bp genome sequence provides opportunities to investigate the biology of a poorly understood lineage within the *Bacteroidetes*.

## ANNOUNCEMENT

LSUCC0859 was isolated from Bay de Pomme d’Or (Louisiana, Gulf of Mexico; 29.35°N, −89.54°W) in January 2017 using dilution-to-extinction (DTE) cultivation with artificial seawater (1, 2). Source water salinity and temperature were 11.7 and 12.38°C, respectively (2). The 16S rRNA gene was previously Sanger sequenced (GenBank accession number MK603739.1) (2), and a BLASTn search of the NCBI nt database found that the closest related cultured organisms were Bizionia psychrotolerans strain sea water13 (99.17% identity; MT112327.1) and *Flavobacteria* bacterium Yb008 (99.17% identity; AB496663.1). However, there were no close hits to genome-sequenced organisms. We therefore sequenced the genome of LSUCC0859 because of its presence within an understudied group of *Flavobacteriaceae*.

In preparation for genome sequencing, one LSUCC0859 cryostock was inoculated into 50 mL MWH3 medium (2) and grown at 25°C for 4 days until the cell density reached ~10^6^ cells mL^−1^. This seed culture was subsequently transferred into three 300-mL and two 600-mL replicates in polycarbonate flasks. After 2 days of growth, cultures reached cell densities of ~1.5 × 10^6^ cells mL^−1^, after which they were each vacuum filtered through a 0.2-μm polyethersulfone filter (Millipore Sigma, USA). A combination of proteinase K digestion, phenol-chloroform-isoamyl alcohol extraction, and ethanol precipitation (https://dx.doi.org/10.17504/protocols.io.b5iiq4ce) was performed to obtain genomic DNA that was quantified using a Qubit fluorometer. Library preparation and sequencing were performed at the Microbial Genome Sequencing Center (https://www.migscenter.com/). Illumina sequencing libraries were constructed with an Illumina DNA prep kit and integrated DNA technologies 10-bp unique dual indexes. Libraries were sequenced on an Illumina NextSeq 2000, producing 2,879,268 2 × 151-bp read pairs, and demultiplexed, adaptor trimmed, and quality controlled with bcl2fastq (v2.20.0422) (3).

We trimmed paired-end sequence data with Trimmomatic v0.38 (4) using LEADING:20 TRAILING:20 SLIDINGWINDOW:13:20 MINLEN:40 and assembled the reads with SPAdes v3.13.0 (5). Scaffolds of <500 bp were manually removed. We mapped raw reads to the genome using the Burrows-Wheeler Aligner (BWA) v0.7.17 (r1188) (6) and samtools v18.0.4 (7) for polishing with Pilon v1.22 (8). The final assembly resulted from the Pilon output. We also used CheckM v1.1.3 (9) for additional quality control, and the genome was annotated using the Prokaryotic Genome Annotation Pipeline (PGAP) (10). Whole-genome taxonomy was performed with GTDB-tk v1.5.0 and the release 202 database using classify_wf (11). Default settings were used for all software unless otherwise noted.

**FIG 1:**
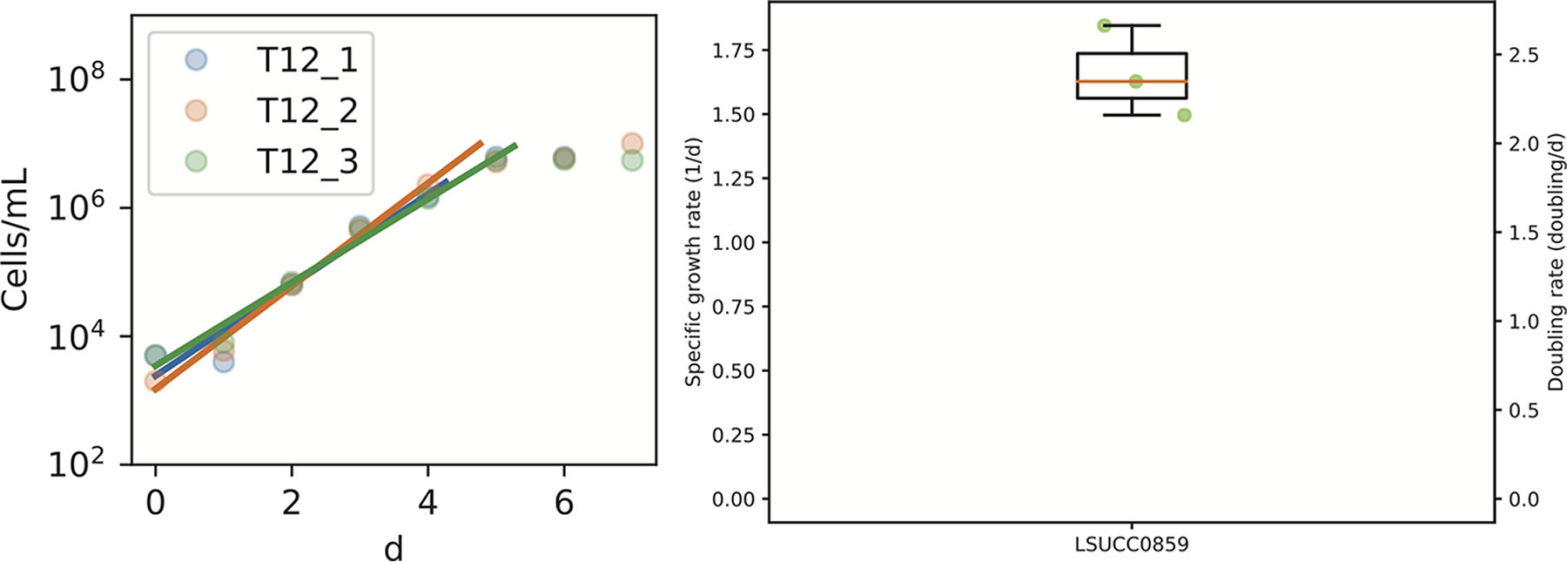
Growth curves (left) and calculated growth rates (right) for LSUCC0859 growing at room temperature on MWH3 medium. Output is from sparse-growth-curve (12).

The draft genome was 2,168,862 bp long in 12 scaffolds (*N*_50_, 468,311 bp), with a GC content of 40.16% and mean coverage of 201×. Of the 1,919 predicted genes, 1,870 were protein coding, and 1 each of the 5S, 16S, and 23S rRNA genes. CheckM estimated that the genome coding density was 93.39%, with 96.26% completion and 0% contamination. GTDB-tk placed the LSUCC0859 genome within the *Flavobacteriaceae*, with the nearest neighbor being the metagenome-assembled genome (MAG) “MAG-120531 sp000173115” (average nucleotide identity, 78.71%). The organism had a mean growth rate of 2.4 doublings day^−1^ (calculated as described in reference 12) at room temperature in MWH3 medium (Fig. 1).

### Data availability.

The accession numbers PRJNA804496, JAKOGP000000000, and SRX14114391 are the NCBI BioProject, GenBank, and SRA accession numbers, respectively. The genome version described in this paper is version JAKOGP010000000. Live cultures or cryostocks of the organism will be provided upon request.

## References

[1] Henson MW, Lanclos VC, Pitre DM, Weckhorst JL, Lucchesi AM, Cheng C, Temperton B, Thrash JC. 2020. Expanding the diversity of bacterioplankton isolates and modeling isolation efficacy with large scale dilution-to-extinction cultivation. Appl Environ Microbiol 86:e00943-20. doi:10.1128/AEM.00943-20. 32561583 PMC7440811

[2] Henson MW, Pitre DM, Weckhorst JL, Lanclos VC, Webber AT, Thrash JC. 2016. Artificial seawater media facilitate cultivating members of the microbial majority from the Gulf of Mexico. mSphere 1:e00028-16. doi:10.1128/mSphere.00124-16. PMC4894692 27303734

[3] Illumina. bcl2fastq: a proprietary Illumina software for the conversion of bcl files to basecalls. https://support.illumina.com/sequencing/sequencing_software/bcl2fastq-conversion-software.html.

[4] Bolger AM, Lohse M, Usadel B. 2014. Trimmomatic: a flexible trimmer for Illumina Sequence Data. Bioinformatics 30:2114–2120. doi:10.1093/bioinformatics/btu170. 24695404 PMC4103590

[5] Prjibelski A, Antipov D, Meleshko D, Lapidus A, Korobeynikov A. 2020. Using SPAdes de novo assembler. Curr Protoc Bioinformatics 70:e102. doi:10.1002/cpbi.102. 32559359

[6] Li H, Durbin R. 2009. Fast and accurate short read alignment with Burrows-Wheeler transform. Bioinformatics 25:1754–1760. doi:10.1093/bioinformatics/btp324. 19451168 PMC2705234

[7] Li H, Handsaker B, Wysoker A, Fennell T, Ruan J, Homer N, Marth G, Abecasis G, Durbin R, 1000 Genome Project Data Processing Subgroup. 2009. The Sequence Alignment/Map format and SAMtools. Bioinformatics 25:2078–2079. doi:10.1093/bioinformatics/btp352. 19505943 PMC2723002

[8] Walker BJ, Abeel T, Shea T, Priest M, Abouelliel A, Sakthikumar S, Cuomo CA, Zeng Q, Wortman J, Young SK, Earl AM. 2014. Pilon: an integrated tool for comprehensive microbial variant detection and genome assembly improvement. PLoS One 9:e112963. doi:10.1371/journal.pone.0112963. 25409509 PMC4237348

[9] Parks DH, Imelfort M, Skennerton CT, Hugenholtz P, Tyson GW. 2015. Assessing the quality of microbial genomes recovered from isolates, single cells, and metagenomes. Genome Res 25:1043–1055. doi:10.1101/gr.186072.114. 25977477 PMC4484387

[10] Tatusova T, DiCuccio M, Badretdin A, Chetvernin V, Nawrocki EP, Zaslavsky L, Lomsadze A, Pruitt KD, Borodovsky M, Ostell J. 2016. NCBI Prokaryotic Genome Annotation Pipeline. Nucleic Acids Res 44:6614–6624. doi:10.1093/nar/gkw569. 27342282 PMC5001611

[11] Chaumeil P-A, Mussig AJ, Hugenholtz P, Parks DH. 2019. GTDB-Tk: a toolkit to classify genomes with the Genome Taxonomy Database. Bioinformatics 36:1925–1927. doi:10.1093/bioinformatics/btz848. PMC7703759 31730192

[12] Cheng C, Thrash JC. 2021. sparse-growth-curve: a computational pipeline for parsing cellular growth curves with low temporal resolution. Microbiol Resour Announc 10:e00296-21. doi:10.1128/MRA.00296-21. 33986091 PMC8142577

